# A High-Protein and Low-Glycemic Formula Diet Improves Blood Pressure and Other Hemodynamic Parameters in High-Risk Individuals

**DOI:** 10.3390/nu14071443

**Published:** 2022-03-30

**Authors:** Martin Röhling, Kerstin Kempf, Winfried Banzer, Klaus Michael Braumann, Dagmar Führer-Sakel, Martin Halle, David McCarthy, Stephan Martin, Jürgen Scholze, Hermann Toplak, Aloys Berg, Hans-Georg Predel, ACOORH Study Group

**Affiliations:** 1 West-German Center of Diabetes and Health, Düsseldorf Catholic Hospital Group, Hohensandweg 37, 40591 Düsseldorf, Germany; kerstin.kempf@wdgz.de (K.K.); stephan.martin@vkkd-kliniken.de (S.M.); 2 Department of Sports Medicine, Institute for Sports and Sport Science, University of Frankfurt, 60487 Frankfurt, Germany; banzer@med.uni-frankfurt.de; 3 Department of Sports and Movement Medicine, Faculty of Psychology and Human Movement Sciences, University of Hamburg, 20148 Hamburg, Germany; braumann@uni-hamburg.de; 4 Diabetes and Metabolism and Division of Laboratory Research, Department of Endocrinology, University Hospital Essen, University Duisburg-Essen, 45122 Essen, Germany; dagmar.fuehrer-sakel@uk-essen.de; 5 Department of Prevention, Rehabilitation and Sports Medicine, Klinikum Rechts der Isar, Technical University of Munich (TUM), 80992 Munich, Germany; martin.halle@mri.tum.de; 6 DZHK (German Centre for Cardiovascular Research), Partner Site Munich Heart Alliance, 80802 Munich, Germany; 7 Public Health Nutrition Research Group, London Metropolitan University, London N7 8DB, UK; d.mccarthy@londonmet.ac.uk; 8 Faculty of Medicine, Heinrich Heine University Düsseldorf, 40591 Düsseldorf, Germany; 9 Kardios Cardiologists in Berlin, 10787 Berlin, Germany; schenkenberger@klinische-forschung-berlin.de; 10 Department of Medicine, Division of Endocrinology and Diabetology, Medical University of Graz, 8010 Graz, Austria; hermann.toplak@medunigraz.at; 11 Faculty of Medicine, University of Freiburg, 79117 Freiburg, Germany; berg.aloys@web.de; 12 Institute of Cardiovascular Research and Sports Medicine, German Sport University Cologne, 50933 Cologne, Germany; predel@dshs-koeln.de

**Keywords:** blood pressure, insulin, lifestyle intervention, formula diet, cardiac autonomic regulation, pulse wave velocity, heart rate

## Abstract

Low-caloric formula diets can improve hemodynamic parameters of patients with type 2 diabetes. We, therefore, hypothesized that persons with overweight or obesity can benefit from a high-protein, low-glycemic but moderate-caloric formula diet. This post-hoc analysis of the Almased Concept against Overweight and Obesity and Related Health Risk- (ACOORH) trial investigated the impact of a lifestyle intervention combined with a formula diet (INT, *n* = 308) compared to a control group with lifestyle intervention alone (CON, *n* = 155) on hemodynamic parameters (systolic and diastolic blood pressure (SBP, DBP), resting heart rate (HR), and pulse wave velocity (PWV)) in high-risk individuals with prehypertension or hypertension. INT replaced meals during the first 6 months (1 week: 3 meals/day; 2–4 weeks: 2 meals/day; 5–26 weeks: 1 meal/day). Study duration was 12 months. From the starting cohort, 304 (68.3%, INT: *n* = 216; CON: *n* = 101) participants had a complete dataset. Compared to CON, INT significantly reduced more SBP (−7.3 mmHg 95% CI [−9.2; −5.3] vs. −3.3 mmHg [−5.9; −0.8], *p* < 0.049) and DBP (−3.7 mmHg [−4.9; −2.5] vs. −1.4 mmHg [−3.1; 0.2], *p* < 0.028) after 12 months. Compared to CON, INT showed a pronounced reduction in resting HR and PWV after 6 months but both lost significance after 12 months. Changes in SBP, DBP, and PWV were significantly associated positively with changes in body weight and fat mass (all *p* < 0.05) and resting HR correlated positively with fasting insulin (*p* < 0.001) after 12 months. Combining a lifestyle intervention with a high-protein and low-glycemic formula diet improves hemodynamic parameters to a greater extent than lifestyle intervention alone in high-risk individuals with overweight and obesity.

## 1. Introduction

Patients with type 2 diabetes or metabolic syndrome are characterized by an increased accompanying risk for hypertension [1], cardiac autonomic neuropathy [2], and/or arterial stiffness [3]. Although intensively treated with antidiabetic, antilipidemic, and antihypertensive pharmacotherapy, patients with type 2 diabetes or metabolic syndrome are inherently at an increased risk for cardiovascular disease (CVD) as well as events (CVE) [4], and life expectancy is decreased [5]. A hypercaloric-high-carbohydrate dietary pattern, leading to hyperinsulinemia and ultimately to obesity, has been associated with this detrimental glucometabolic and cardiovascular state [6,7]. Increased chronic insulin stimulation, together with the accompanying lipogenesis and ectopic fat formation also leads to disadvantageous remodeling processes in organs, as well as in the vascular and nervous system [8].

Numerous studies and reviews have shown that cardiovascular risk factors can be improved by lifestyle modification [9,10]. A recently published weight management study incorporating a low-caloric formula diet demonstrated that patients with type 2 diabetes can benefit from weight loss in the long term with respect to reduction in blood pressure and antihypertensive medications [11]. The extent to which this benefit occurs in persons with overweight or obesity and at least one further co-morbidity of the metabolic syndrome is yet unknown. Equally, the impact of elevated fasting insulin levels as a possible initial cause of obesity and type 2 diabetes has not been considered in the context of cardiovascular risk factors and meal replacement-based interventions.

The multicenter, international and randomized controlled ‘Almased Concept against Overweight and Obesity and Related Health Risk’ (ACOORH)-trial was initiated to investigate the additional effect of a low-carbohydrate formula diet on top of a low-intensity lifestyle intervention in comparison to a lifestyle intervention alone in a larger cohort of high-risk individuals with overweight or obesity and at least one further co-morbidity of the metabolic syndrome. Previously published works of the ACOORH trial have already shown a beneficial effect on the prediabetes conversion rate to normoglycemia [12], weight loss [13] and nutritional behavior [14], as well as glucometabolic and inflammatory markers [15]. The present study analyzed the changes in hemodynamic parameters after 6 and 12 months.

## 2. Materials and Methods

### 2.1. Study Design and Population

Study design and population has been described in detail elsewhere [12,13]. Briefly, volunteers (*n* = 463, age = 51 ± 10 years, sex [male] = 36%, BMI = 31.6 ± 2.4 kg/m^2^) were randomized with a ratio of 1:2 into either a low-intensive lifestyle intervention group (CON, *n* = 155) or a low-intensive lifestyle intervention group which was combined with a structured meal replacement regime (INT, *n* = 308). Study duration was 12 months and the intensive meal replacement intervention phase was conducted within the first 6 months and follow-up was after 6 months. This international trial received ethical approval (registered at drks.de; ID: DRKS00006811) for each participating center. Study reporting adhered to CONSORT guidelines. All participants provided written informed consent. Inclusion and exclusion criteria have been described in detail elsewhere [13]. Briefly, participants were included when they were free of diabetes; aged 21–65 years; had a BMI of 27–35 kg/m^2^; waist circumference ≥88 cm (≥102 cm) for females (males); in addition, at least one further co-morbidity of the metabolic syndrome had to be present.

### 2.2. Intervention and Meal Replacement Regimen

A detailed description of the intervention program and meal replacement regimen has been published elsewhere [13]. Briefly, at the beginning of the study, both CON and INT were provided with information about a healthy lifestyle (e.g., healthy cooking, physical activity, a guideline booklet for behavior changing) during a lifestyle counselling session and were encouraged to lose weight. Additionally, both groups were provided with telemetric scales (smartLAB scale W; HMM Holding AG, Dossenheim, Germany) and pedometers (smartLAB walk P+; HMM Holding AG, Dossenheim, Germany). Prior to each study visit, participants completed a 4-day, unweighed diet record. All behavioral records (i.e., diet and physical activity) were discussed at each study visit.

INT underwent an accompanying high-protein, low-glycemic, and moderate-caloric meal replacement regimen with a liquid soy-yogurt-honey-based formula diet (Almased-Vitalkost^®^, Almased-Wellness-GmbH, Oberding, Germany [16]) during the first 6 months of the intervention. This formula diet-based meal replacement regimen was paralleled with a stepwise reintroduction of regular foods typically of a low-carbohydrate dietary approach. Management of the formula diet regime has been described in detail elsewhere [13].

### 2.3. Measurements

All measurements were performed at baseline as well as after 6, and 12 months. Laboratory data (fasting insulin, fasting glucose, and HbA1c) were collected as described in detail elsewhere [13]. Body composition (fat mass, fat-free mass, BMI, and weight) was determined by using a medical body composition analyzer (Seca medical Body Composition Analyzer^®^ (seca-mBCA 115), Hamburg, Germany [17]). Hemodynamic parameters (systolic blood pressure (SBP), diastolic blood pressure (DBP), resting heart rate (HR), and pulse wave velocity (PWV)) were measured by using a validated device (Mobil-O-Graph PWA; I.E.M. GmbH, Stolberg, Germany [18]) with an appropriately sized cuff. First-phase systolic and fifth-phase diastolic Korotkoff blood pressure were auscultated from the right arm of each participant, after resting 5 min and sitting upright. Two measurements were performed and the average of these measurements were used as the final result for blood pressure, resting HR, and PWV. An external monitor supervised and reviewed documentation regarding adverse and serious adverse events [19].

### 2.4. Statistics

Sample size calculation and inherent assumptions have been described in detail elsewhere [13]. The primary outcome of the original ACOORH study was the change in body weight between INT and CON following the intervention [13]. In this post-hoc analysis, the primary outcome was the change in hemodynamic parameters (SBP, DBP, HR, and PWV) after 6 and 12 months. Risk groups for these four parameters were defined as follows. SBP and DBP were redefined according to the current guidelines for grading blood pressure into hypertensive (≥140 mmHg; ≥90 mmHg), prehypertensive (121–139 mmHg; 81–89 mmHg) and normotensive stages (≤120 mmHg; ≤80 mmHg) [20]. Resting HR and PWV risk groups were defined by stratifying patients into tertiles and the upper 2 tertiles were assumed to have an inherent higher risk in comparison to the lowest tertile. All performed intra and intergroup comparisons focused on the predefined high-risk groups and based on multivariable linear regression analyses. Furthermore, hemodynamic parameters were analyzed for associations with anthropometric and laboratory parameters to determine any physiological interrelationships. Statistical analysis was performed by an independent institute (ACOMED statistik^®^, Leipzig, Germany) and a detailed description about handling parametric and non-parametric data, missing values ((per-protocol (PP) and intention-to-treat (ITT) analysis, last-observation-carried-forward (LOCF) method), and applied software can be found elsewhere [13]. All statistical tests were two-sided, significant results were assumed to be *p* < 0.05 and if not otherwise stated, ITT analysis findings were reported.

## 3. Results

**Figure 1:**
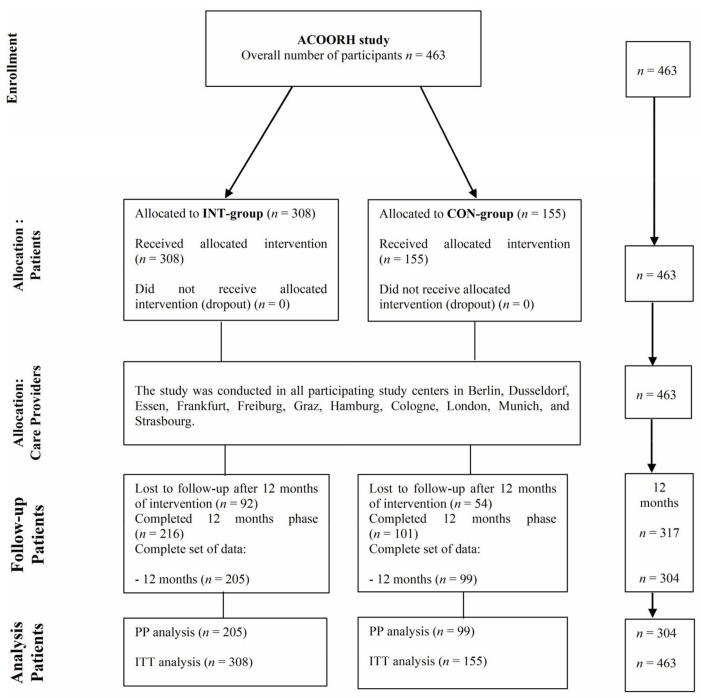
Flowchart. ACOORH, Almased Concept against Overweight and Obesity and Related Health Risk; CON, control group; INT, intervention group; ITT, intention-to-treat analysis; PP, per protocol analysis.

Three hundred and seventeen of 463 participants (68.3%, INT: *n* = 216; CON: *n* = 101) completed the study after 12 months. Baseline characteristics of the whole cohort (*n* = 463) can be found elsewhere [13]. A complete set of data were available for *n* = 304 participants (65.7%, INT: *n* = 205; CON: *n* = 99) (Figure 1) in the present study.

Anthropometric, laboratory and hemodynamic parameters at baseline are displayed in Table 1. Reasons for dropouts have been presented elsewhere [13]. Frequencies of antihypertensive drugs did not differ between INT and CON at baseline (Supplementary Materials Table S1) and remained unaltered during the 12-months study period. A post-hoc reallocation into risk subgroups is displayed in Supplementary Materials Figure S1 and was performed in order to identify hemodynamic parameter-based high-risk groups.

### 3.1. Intra and Intergroup Differences in Hemodynamic Parameters after 6 and 12 Months Focusing on Risk Groups

Prehypertensive and hypertensive participants of INT significantly improved SBP and DBP after 6 and 12 months. CON participants with prehypertension and hypertension primarily reduced SBP and DBP after 6 months, but this significance diminished after 12 months (Table 2). Intergroup comparisons showed a significant difference in DBP (INT: −4.6 mmHg 95% CI [−5.7; −3.2] vs. CON: −1.6 mmHg 95% CI [−3.3; −0.1], *p* < 0.009) after 6 months and in SBP (INT: −7.3 mmHg 95% CI [−9.2; −5.3] vs. CON: −3.3 mmHg 95% CI [−5.9; −0.8], *p* < 0.049) and DBP (INT: −3.7 mmHg 95% CI [−4.9; −2.5] vs. CON: −1.4 mmHg 95% CI [−3.1; 0.2], *p* < 0.028) after 12 months, indicating a moderate effect for the INT group (Supplementary Materials Figure S1).

Resting HR was significantly reduced in both groups, particularly in INT, after 6 and 12 months (Table 2). Compared to CON, INT showed a significant reduction in resting HR after 6 months. This intergroup difference diminished after 12 months.

Compared to CON, INT showed a significant reduction in PWV after 6 months and this improvement remained significant after 12 months (Table 2). However, the intergroup difference diminished after 12 months.

### 3.2. Association of Changes in Hemodynamic Parameters with Clinical Parameters

According to the whole cohort with a complete dataset, changes in SBP were significantly positively correlated with changes in body weight and fat mass after 6 and 12 months (all *p* < 0.001), even after Bonferroni correction for multiple testing and adjustment for age, sex and BMI (Table 3). Changes in DBP were also significantly associated positively with changes in body weight and fat mass after 6 (both *p* < 0.001) and 12 months (both *p* < 0.05) of intervention. These associations lost significance after Bonferroni correction and adjustment for age, sex, and BMI. Changes in resting HR were significantly positively associated with changes in fasting insulin after 6 and 12 months of intervention (both *p* < 0.001), and these associations remained significant even after Bonferroni correction and adjustment for age, sex, and BMI (Table 3). Changes in PWV were significantly positively correlated with changes in body weight and fat mass after 6 months of intervention. This correlation remained only significant in body weight when adjusted for multiple testing and the covariables of age, sex, and BMI (*p* < 0.001) (Table 3).

**Figure 2:**
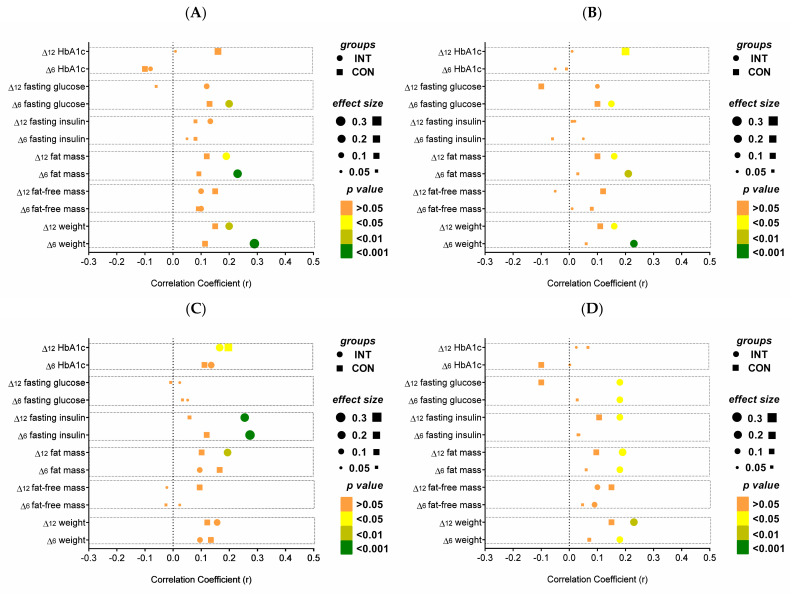
Correlation of changes in clinical parameters with changes in (**A**) SBP, (**B**) DBP, (**C**) resting HR, and (**D**) PWV after 6 and 12 months of intervention. Effect size and significance are shown for INT and CON. Δ6 = change after 6 months; Δ12 = change after 12 months. CON, control group; DBP, diastolic blood pressure; HbA1c, hemoglobin A1c; HR, resting heart rate; INT, intervention group; PWV, pulse wave velocity; SBP, systolic blood pressure.

The aforementioned associations were even stronger for the INT group when considering group allocation (Figure 2). In particular, the association of (1) SBP with body weight (r = 0.291) or fat mass (r = 0.239); and (2) DBP with body weight (r = 0.237) or fat mass (r = 0.219) indicated stronger correlations after 6 months between INT and CON; (3) Correlation of resting HR with fasting insulin (r = 0.232) showed pronounced effect sizes for INT compared to CON after 6 and 12 months.

Further subgroup analyses revealed that in particular reductions in SBP and DBP were attributed to changes in weight loss showing large effect sizes in the prehypertensive and hypertensive groups (Supplementary Materials Table S2). INT and CON showed significant body weight reductions from baseline (T0) to 6 (T1) and 12 (T2) months in hypertensive (INT: T0: 94 ± 17 kg, T1: 85 ± 16 kg, T2: 88 ± 17 kg; CON: T0: 91 ± 10 kg, T1: 85 ± 11 kg, T2: 86 ± 12 kg) and prehypertensive participants (INT: T0: 93 ± 13 kg, T1: 85 ± 13 kg, T2: 88 ± 14 kg; CON: T0: 95 ± 13 kg, T1: 91 ± 13 kg, T2: 92 ± 14 kg) (Supplementary Materials, Table S2).

**Figure 3:**
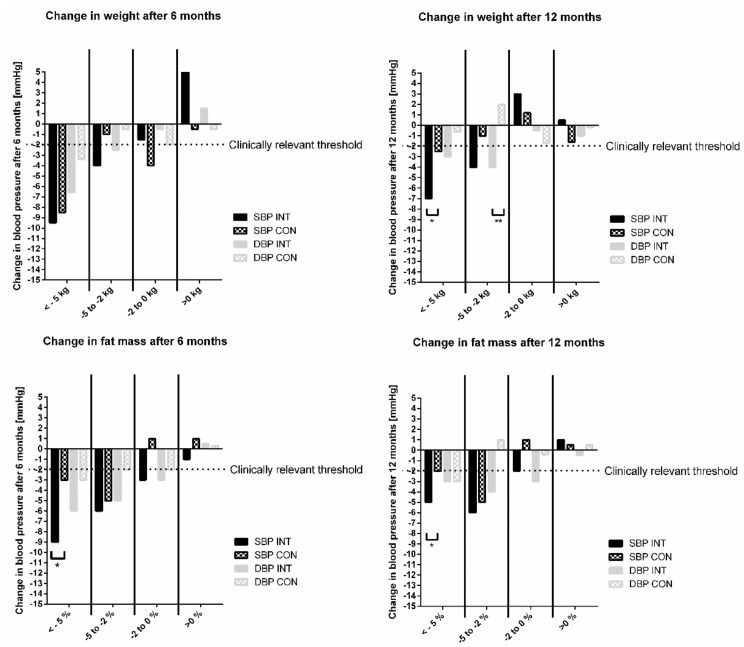
Clinically relevant blood pressure reduction due to weight and fat mass loss after 6 and 12 months. Data are shown as mean or percentages. ** *p* < 0.01; * *p* < 0.05. CON, control group; DBP, diastolic blood pressure; INT, intervention group; SBP, systolic blood pressure.

From a clinical perspective, without considering group allocation, a clinically relevant improvement of hemodynamic parameters (e.g., reduction in SBP or DBP of ≥2 mmHg [20,21]) occurred when participants lost more than 2 kg body weight or more than 2% fat mass following the intervention (Figure 3).

## 4. Discussion

The results of this post-hoc analysis of the ACOORH trial indicate larger reductions in systolic and diastolic blood pressure as well as improvements in resting heart rate and pulse wave velocity after a 12-month intervention with a high-protein, low-glycemic, and moderate-caloric formula diet in high-risk individuals with overweight or obesity and accompanied cardiovascular risk factors compared to a low-intensity lifestyle intervention alone.

These findings are in line with other studies investigating the impact of weight loss due to different dietary and lifestyle approaches on cardiovascular risk factors in various populations [9,22,23]. For example, calorie restricted diets [22] or time-restricted eating approaches [23] have led to significant reductions in blood pressure and improvements of endothelial dysfunction. Furthermore, a recently published meta-analysis demonstrated comparable improvements in cardiovascular risk factors following weight loss intervention with diets of different macronutrient composition [9], pointing towards a more pronounced effect for improving systolic and diastolic blood pressure following a lower carbohydrate diet (e.g., Atkins diet). Moreover, adding a moderate-caloric restrictive diet to an aerobic exercise intervention demonstrated superior improvements in proximal aortic stiffness in older adults with obesity compared to exercise alone [24].

Although high-risk individuals of the INT group demonstrated moderate but clinically meaningful improvements in hemodynamic parameters, CON participants also showed clinically relevant changes. These small differences between both groups might have been derived from the dietary composition throughout the study. As recently published [25], both groups differed primarily in protein consumption which was embedded in a prescribed low-carbohydrate dietary lifestyle for both intervention groups. This explanation can be supported by a recently published meta-analysis showing the beneficial effects of higher compared to lower protein diets on cardiometabolic risk factors [26]. The mechanistic link behind this finding might have been derived from bioactive peptides that can inhibit the activity of the renin-angiotensin converting enzyme which is a key regulator for systemic hypertension [27].

Findings of the correlation analyses of the whole cohort pointed towards physiological interrelationships between changes in fat mass and body weight with blood pressure and pulse wave velocity as well as interactions of fasting insulin and resting heart rate. These different associations may derive directly or indirectly from one pathophysiological mechanism of hyperinsulinemia or higher insulin levels leading to several further physiological maladaptive changes, for example: (1) sympathetic nervous system overactivity [28]; (2) enhanced renal sodium reabsorption [8]; (3) overstimulated renin-angiotensin-aldosterone system [8]; (4) proinflammatory processes and hypertrophy of vascular smooth muscle cells (or endothelial dysfunction and increased arterial stiffness) [28]; and finally (5) increases in body weight and obesity-associated hypertension [29]. One further key player in this context is insulin resistance, which in combination with hyperinsulinemia, may promote hypertension and atherogenesis. During insulin resistance, nitric oxide (NO) production is impaired while the supportive effect of insulin on calcium ion influx and vasoconstriction is still present [8]. Furthermore, adipokines—segregated by adipose tissue, impair the regulation of blood pressure, lipid and glucose metabolism [30] and changes in adipokine levels might have contributed to the improvement of hemodynamic parameters in the present study.

The strong positive correlation of resting HR with fasting insulin levels in the present study is in line with other studies demonstrating that insulin itself (even in different entities such as: fasting insulin, intact proinsulin, split proinsulin, or acute insulin response [31]) can influence the cardiac autonomic nervous system by reducing parasympathetic function and potentiating sympathetic drive in patients with [32] and without diabetes [33].

When discussing the sustainability of the intervention effects, changes of SBP and DBP, which were primarily associated with changes in weight and/or fat mass, seem to be more long-lasting than resting HR and PWV. Resting HR was, especially, probably more related to short-term physiological changes of insulin, which have been shown to be present in particular during the intensive phase of the study [15]. The primary intervention was within the first 6 months (intensive phase) and the last 6 months were characterized as a maintain phase (less intensive phase). The decline in treatment intensity maybe led to this immediate physiological adaption of insulin, also influencing negatively the sympathetic nervous system while weight loss and/or fat mass reduction was more sustainable, and, therefore, SBP and DBP were significantly different between both groups even after 12 months of intervention. The landmark studies of DiRECT [11] and DIADEM-1 [34] have already shown in patients with type 2 diabetes that, in particular, weight-loss mediates the reduction of blood pressure.

Frequency of antihypertensive medication use was not affected by the intervention in either group. One possible explanation for these findings is, that, on the one hand, compared to very-low or low-caloric diet interventions with partial abandonment of antihypertensive medication [11,35], the present study applied a moderate-caloric diet (≈1300–1500 kcal per day) approach, with the aim of increasing study and treatment adherence and reducing the risk for adverse outcomes in the long-term [36]. On the other hand, reduction of antihypertensive medication was not targeted and not all participants were hypertensive or prehypertensive with an accompanying antihypertensive therapy.

The strengths of the present post-hoc analysis comprise a large sample size of only high-risk participants with overweight or obesity and at least one additional co-morbidity of the metabolic syndrome, the international, multicenter and randomized controlled setting of the study, and a relatively long study period over 12 months. Although study participants were comprehensively characterized, information about smoking status as well as kidney-related data (e.g., creatinine levels or urinary sodium excretion) were not included, which should be considered when interpreting the data. A recently published meta-analysis demonstrated that a reduction in dietary sodium can have beneficial effects on blood pressure levels in different populations [37]. Moreover, it has been shown that cardiorespiratory fitness also has a marked impact on the autonomic nervous system in patients with and without diabetes [38,39]. In particular, parasympathetic drive increases with enhancing cardiorespiratory fitness. This aspect was not part of the investigation and should be considered when interpreting the data. Further limitations of the present study include that there was no constant diet monitoring. Based on the issue of biased dietary records with systematic errors, we chose to omit monitoring the participants’ diets [40]. However, 4-day diet diaries prior to each study visit were required from all participants to support the lifestyle counseling. Furthermore, although sophisticated imputation methods for missing values are currently indicated, we consciously chose the LOCF procedure for a more conservative statistical approach to prevent overestimating the present results of this post-hoc analysis. Another aspect which needs to be considered, especially when discussing the changes to the resting HR data, is that there is a possibility of an unconscious habituation towards the examination procedure which might have influenced the measurements. However, randomization into one of the two groups should have abolished any potential habituation effect, particularly because baseline characteristics of both groups were not different. On the other hand, the strong interrelation between fasting insulin and resting HR at any time point, particularly for the INT group, indicates rather a physiological adaption of resting HR due to the intervention.

## 5. Conclusions

Taken together, a low-intensity lifestyle intervention combined with a high-protein, low-glycemic, and moderate-caloric formula diet led to a clinically relevant outcome regarding hemodynamic parameters in high-risk individuals with overweight and obesity and at least one further factor of the metabolic syndrome. The present therapeutic approach combining lifestyle interventions with an accompanying formula diet regimen should be considered as a valid option for the management of cardiovascular risk factors which are related to overweight and obesity. From a clinical perspective, health care providers should encourage patients with hypertension to lose weight greater than 2 kg and also follow a lifestyle that prevents chronic elevated insulin levels and cardiac autonomic dysregulation.

## Figures and Tables

**Table 1:** Baseline characteristics of the participants completing the whole study.

	INT-Group (*n* = 205)	CON-Group (*n* = 99)
Clinical parameters		
Sex (% male)	35.2	40.6
Age (years)	51 ± 10	50 ± 9
Weight (kg)	92 ± 14	94 ± 12
BMI (kg/m^2^)	31.5 ± 2.3	31.5 ± 2.4
Fat mass (kg)	36.6 ± 6.6	37.2 ± 6.5
Fat-free mass (kg)	54.9 ± 11.7	56.4 ± 11.5
Fasting insulin (µU/mL)	15.9 ± 10.3	15.1 ± 8.7
Fasting glucose (mg/dL)	94 ± 12	93 ± 11
HbA1c (%)	5.50 ± 0.34	5.47 ± 0.38
Hemodynamic parameters		
SBP (mmHg)		
Normotension (≤120 mmHg)	113 ± 6 (*n* = 86)	112 ± 6 (*n* = 44)
Prehypertension (121–139 mm Hg)	129 ± 6 (*n* = 91)	128 ± 5 (*n* = 40)
Hypertension (≥140 mmHg)	152 ± 11 (*n* = 28)	156 ± 11 (*n* = 15)
DBP (mmHg)		
Normotension (≤80 mmHg)	64 ± 15 (*n* = 59)	64 ± 18 (*n* = 26)
Prehypertension (81–89 mm Hg)	85 ± 2 (*n* = 53)	85 ± 2 (*n* = 31)
Hypertension (≥90 mmHg)	99 ± 8 (*n* = 93)	97 ± 5 (*n* = 42)
Resting HR (bpm)		
1st tertile	60 ± 6 (*n* = 68)	60 ± 5 (*n* = 39)
2nd tertile	71 ± 3 (*n* = 70)	71 ± 2 (*n* = 24)
3rd tertile	84 ± 7 (*n* = 67)	83 ± 7 (*n* = 36)
PWV (m/s)		
1st tertile	6.4 ± 0.7 (*n* = 68)	6.3 ± 0.6 (*n* = 34)
2nd tertile	7.7 ± 0.3 (*n* = 67)	7.7 ± 0.3 (*n* = 35)
3rd tertile	8.9 ± 0.6 (*n* = 70)	8.9 ± 0.7 (*n* = 30)

Data are presented as means ± standard deviations, or percentages. BMI, body mass index; DBP, diastolic blood pressure; HR, resting heart rate; PWV, pulse wave velocity; SBP, systolic blood pressure.

**Table 2:** Intra and intergroup changes in the INT and CON-group after 6 and 12 months compared to baseline focusing on individual risk groups.

	Baseline		6 Months			12 Months		
	INT	CON	INT	CON	P (INT vs. CON)	INT	CON	P (INT vs. CON)
SBP (mmHg) ^‡^								
ITT (*n* = 180 vs. 89)	134 ± 11	133 ± 10	−8.6 [−11.1; −7.3] ***	−7.9 [−10.9; −5.4] ***	0.652	−7.3 [−9.2; −5.3] ***	−3.3 [−5.9; −0.8] *	0.049
PP (*n* = 119 vs. 55)	134 ± 12	133 ± 11	−10.6 [−11.4; −9.6] ***	−9.9 [−13.7; −6.3] ***	0.534	−8.8 [−11.4; −6.1] ***	−4.0 [−7.9; −0.1] *	0.031
DBP (mmHg) ^†^								
ITT (*n* = 225 vs. 117)	94 ± 10	92 ± 8	−4.6 [−5.7; −3.2] ***	−1.6 [−3.3; −0.1] *	0.009	−3.7 [−4.9; −2.5] ***	−1.4 [−3.1; 0.2]	0.028
PP (*n* = 146 vs. 73)	94 ± 9	92 ± 7	−5.8 [−7.0; −4.1] ***	−2.1 [−4.1; −0.2] *	0.003	−4.4 [−5.8; −3.0] ***	−1.5 [−3.4; 0.5]	0.019
Resting HR (bpm) ^$^								
ITT (*n* = 208 vs. 94)	78 ± 8	77 ± 8	−4.0 [−5.3; −2.7] ***	−1.6 [−3.5; 0.4]	0.045	−2.7 [−4.0; −1.4] ***	−2.4 [−4.3; −0.4] *	0.783
PP (*n* = 137 vs. 60)	77 ± 8	78 ± 8	−5.0 [−6.6; −3.4] ***	−3.6 [−6.3; −1.2] **	0.353	−3.4 [−4.9; −1.6] ***	−4.5 [−6.9; −2.3] ***	0.240
PWV (m/s) ^$^								
ITT (*n* = 194 vs. 98)	8.4 ± 0.7	8.2 ± 0.7	−0.18 [−0.28; −0.08] ***	−0.07 [−0.20; 0.13]	0.043	−0.10 [−0.18; −0.03] *	−0.01 [−0.11; 0.10]	0.110
PP (*n* = 137 vs. 65)	8.3 ± 0.7	8.3 ± 0.8	−0.25 [−0.36; −0.16] ***	−0.11 [−0.27; 0.15]	0.049	−0.09 [−0.18; 0.01]	0.02 [−0.12; 0.16]	0.200

Data are shown as mean ± SD and mean [95% CI]. *** *p* < 0.001 vs. baseline; ** *p* < 0.01 vs. baseline; * *p* < 0.05 vs. baseline. ^‡^ hypertensive and prehypertensive participants (>120 mmHg) were considered; ^†^ hypertensive and prehypertensive participants (>80 mmHg) were considered; ^$^ upper two tertiles (with highest values); Differences in changes after 6 and 12 months between both groups were analyzed using ANCOVAs adjusting for baseline values. DBP, diastolic blood pressure; HR, heart rate; ITT, intention to treat; PP, per protocol; PWV, pulse wave velocity; SBP, systolic blood pressure.

**Table 3:** Correlation of changes of hemodynamic and clinical parameters after 6 and 12 months of intervention from all participants completing the study (*n* = 304).

Hemodynamic Parameters Clinical Parameters		Systolic Blood Pressure		Diastolic Blood Pressure		Resting Heart Rate		Pulse Wave Velocity	
		Δ 6 Months	Δ 12 Months	Δ 6 Months	Δ 12 Months	Δ 6 Months	Δ 12 Months	Δ 6 Months	Δ 12 Months
Δ Weight	r	+0.336	+0.206	+0.260	+0.132	+0.186	+0.144	+0.255	+0.198
[kg]	*p*	**<0.001**	**<0.001**	**<0.001**	0.025	0.002	0.015	**<0.001**	**<0.001**
Δ Fat mass	r	+0.290	+0.207	+0.253	+0.155	+0.172	+0.163	+0.213	+0.155
[kg]	*p*	**<0.001**	**<0.001**	**<0.001**	0.008	0.003	0.006	**<0.001**	0.008
Δ Fat-free mass	r	+0.179	+0.170	+0.117	+0.030	+0.019	+0.021	+0.092	+0.180
[kg]	*p*	0.003	0.004	0.049	0.611	0.748	0.723	0.190	0.002
Δ Fasting insulin	r	+0.128	+0.110	+0.036	+0.002	+0.229	+0.220	+0.111	+0.132
[µU/mL]	*p*	0.030	0.062	0.541	0.878	**<0.001**	**<0.001**	0.062	0.025
Δ Glukose	r	+0.123	+0.069	+0.123	+0.046	+0.057	+0.014	+0.119	+0.066
[mg/dL]	*p*	0.030	0.248	0.039	0.440	0.335	0.809	0.045	0.264
Δ HbA1c	r	+0.067	+0.121	+0.052	+0.098	+0.128	+0.134	+0.030	+0.047
[%]	*p*	0.259	0.175	0.380	0.098	0.031	0.003	0.616	0.426

Bold *p* values indicate significance after Bonferroni correction for multiple testing (*p* = 0.001). Underlined *p*-values represent significance after adjustment for age, sex, and BMI. HbA1c, hemoglobin A1c.

## Data Availability

The datasets generated during and/or analyzed during the current study are not publicly available but are available from the corresponding author on reasonable request.

## Supplementary Materials

The following supporting information can be downloaded at: https://www.mdpi.com/article/10.3390/nu14071443/s1, Figure S1: Changes of (A) SBP, (B) DBP, (C) resting HR, and (D) PWV after 6 and 12 months of all participants who finished the study (INT + CON); Figure S2: Changes of (A) SBP, (B) DBP, (C) resting HR, and (D) PWV after 6 and 12 months compared between high-risk individuals of INT and CON; Table S1: Antihypertensive drugs of study completers at baseline compared between INT and CON; Table S2: Correlation of changes in weight and blood pressure (SBP, DBP) stratified by the initial hypertensive, prehypertensive, or normotensive status. Table S3: All contributors from the ACOORH Study Group.

## References

[1] Emdin C.A., Rahimi K., Neal B., Callender T., Perkovic V., Patel A. (2015). Blood pressure lowering in type 2 diabetes: A systematic review and meta-analysis. JAMA.

[2] Jiang X., Liu X., Wu S., Zhang G.Q., Peng M., Wu Y., Zheng X., Ruan C., Zhang W. (2015). Metabolic syndrome is associated with and predicted by resting heart rate: A cross-sectional and longitudinal study. Heart.

[3] Safar M.E. (2018). Arterial stiffness as a risk factor for clinical hypertension. Nat. Rev. Cardiol..

[4] Gaede P., Vedel P., Larsen N., Jensen G.V., Parving H.H., Pedersen O. (2003). Multifactorial intervention and cardiovascular disease in patients with type 2 diabetes. N. Engl. J. Med..

[5] Liu X., Wu S., Song Q., Wang X. (2021). Reversion from Pre-Diabetes Mellitus to Normoglycemia and Risk of Cardiovascular Disease and All-Cause Mortality in a Chinese Population: A Prospective Cohort Study. J. Am. Heart Assoc..

[6] Kolb H., Martin S. (2017). Environmental/lifestyle factors in the pathogenesis and prevention of type 2 diabetes. BMC Med..

[7] Kolb H., Stumvoll M., Kramer W., Kempf K., Martin S. (2018). Insulin translates unfavourable lifestyle into obesity. BMC Med..

[8] Kolb H., Kempf K., Röhling M., Martin S. (2020). Insulin: Too much of a good thing is bad. BMC Med..

[9] Ge L., Sadeghirad B., Ball G.D.C., da Costa B.R., Hitchcock C.L., Svendrovski A., Kiflen R., Quadri K., Kwon H., Karamouzian M. (2020). Comparison of dietary macronutrient patterns of 14 popular named dietary programmes for weight and cardiovascular risk factor reduction in adults: Systematic review and network meta-analysis of randomised trials. BMJ.

[10] Schwingshackl L., Zähringer J., Nitschke K., Torbahn G., Lohner S., Kühn T., Fontana L., Veronese N., Schmucker C., Meerpohl J.J. (2021). Impact of intermittent energy restriction on anthropometric outcomes and intermediate disease markers in patients with overweight and obesity: Systematic review and meta-analyses. Crit. Rev. Food Sci. Nutr..

[11] Leslie W.S., Ali E., Harris L., Messow C.M. (2021). Antihypertensive medication needs and blood pressure control with weight loss in the Diabetes Remission Clinical Trial (DiRECT). Diabetologia.

[12] Röhling M., Kempf K., Banzer W., Berg A., Braumann K.M., Tan S., Halle M., McCarthy D., Pinget M., Predel H.-G. (2020). Prediabetes Conversion to Normoglycemia Is Superior Adding a Low-Carbohydrate and Energy Deficit Formula Diet to Lifestyle Intervention-A 12-Month Subanalysis of the ACOORH Trial. Nutrients.

[13] Halle M., Röhling M., Banzer W., Braumann K.M., Kempf K., McCarthy D., Schaller N., Predel H.-G., Scholze J., Führer-Sakel D. (2021). Meal replacement by formula diet reduces weight more than a lifestyle intervention alone in patients with overweight or obesity and accompanied cardiovascular risk factors-the ACOORH trial. Eur. J. Clin. Nutr..

[14] Röhling M., Stensitzky A., Oliveira C.L.P., Beck A., Braumann K.M., Halle M., Führer-Sakel D., Kempf K., McCarthy D., Predel H.-G. (2021). Effects of a Protein-Rich, Low-Glycaemic Meal Replacement on Changes in Dietary Intake and Body Weight Following a Weight-Management Intervention—The ACOORH Trial. Nutrients.

[15] Kempf K., Röhling M., Banzer W., Braumann K.M., Halle M., McCarthy D., Predel H.-G., Schenkenberger I., Tan S., Toplak H. (2021). High-Protein, Low-Glycaemic Meal Replacement Decreases Fasting Insulin and Inflammation Markers—A 12-Month Subanalysis of the ACOORH Trial. Nutrients.

[16] Koohkan S., McCarthy D., Berg A. (2017). The effect of a soy-yoghurt-honey product on excess weight and related Page health risk factors—A review. J. Nutr. Health Food Sci..

[17] Bosy-Westphal A., Schautz B., Later W., Kehayias J.J., Gallagher D., Müller M.J. (2013). What makes a BIA equation unique? Validity of eight-electrode multifrequency BIA to estimate body composition in a healthy adult population. Eur. J. Clin. Nutr..

[18] Hametner B., Wassertheurer S., Kropf J., Mayer C., Eber B., Weber T. (2013). Oscillometric estimation of aortic pulse wave velocity: Comparison with intra-aortic catheter measurements. Blood Press. Monit..

[19] U.S. Food and Drug Administration (2018). Investigational New Drug Application (IND).

[20] Makai P., IntHout J., Deinum J., Jenniskens K., Wilt G.J.V. (2017). A Network Meta-Analysis of Clinical Management Strategies for Treatment-Resistant Hypertension: Making Optimal Use of the Evidence. J. Gen. Intern. Med..

[21] Whelton P.K., He J., Appel L.J., Cutler J.A., Havas S., Kotchen T.A., Roccella E.J., Stout R., Vallbona C., Winston M.C. (2002). Primary prevention of hypertension: Clinical and public health advisory from The National High Blood Pressure Education Program. JAMA.

[22] Di Daniele N., Marrone G. (2021). Effects of Caloric Restriction Diet on Arterial Hypertension and Endothelial Dysfunction. Nutrients.

[23] Gabel K., Cienfuegos S., Kalam F., Ezpeleta M., Varady K.A. (2021). Time-Restricted Eating to Improve Cardiovascular Health. Curr. Atheroscler. Rep..

[24] Brinkley T.E., Leng I., Bailey M.J., Houston D.K., Hugenschmidt C.E., Nicklas B.J., Hundley W.G. (2021). Effects of Exercise and Weight Loss on Proximal Aortic Stiffness in Older Adults with Obesity. Circulation.

[25] Vogtschmidt Y.D., Raben A., Faber I., de Wilde C., Lovegrove J.A., Givens D.I., Pfeiffer A.F., Soedamah-Muthu S.S. (2021). Is protein the forgotten ingredient: Effects of higher compared to lower protein diets on cardiometabolic risk factors. A systematic review and meta-analysis of randomised controlled trials. Atherosclerosis.

[26] Fekete Á.A., Giromini C. (2016). Whey protein lowers blood pressure and improves endothelial function and lipid biomarkers in adults with prehypertension and mild hypertension: Results from the chronic Whey2Go randomized controlled trial. Am. J. Clin. Nutr..

[27] Fu Q. (2019). Sex differences in sympathetic activity in obesity and its related hypertension. Ann. N. Y. Acad. Sci..

[28] Farkhondeh T., Llorens S. (2020). An Overview of the Role of Adipokines in Cardiometabolic Diseases. Molecules.

[29] Tanaka M. (2020). Improving obesity and blood pressure. Hypertens. Res..

[30] Rabe K., Lehrke M., Parhofer K.G., Broedl U.C. (2008). Adipokines and insulin resistance. Mol. Med..

[31] Festa A., D’Agostino R., Hales C.N., Mykkänen L., Haffner S.M. (2000). Heart rate in relation to insulin sensitivity and insulin secretion in nondiabetic subjects. Diabetes Care.

[32] Manzella D., Paolisso G. (2005). Cardiac autonomic activity and Type II diabetes mellitus. Clin. Sci..

[33] Hansen C.S., Færch K. (2019). Heart Rate, Autonomic Function, and Future Changes in Glucose Metabolism in Individuals without Diabetes: The Whitehall II Cohort Study. Diabetes Care.

[34] Taheri S., Zaghloul H., Chagoury O., Elhadad S., Ahmed S.H., El Khatib N., Amona R.A., El Nahas K., Suleiman N., Alnaama A. (2020). Effect of intensive lifestyle intervention on bodyweight and glycaemia in early type 2 diabetes (DIADEM-I): An open-label, parallel-group, randomised controlled trial. Lancet Diabetes Endocrinol..

[35] Leslie W.S., Taylor R., Harris L., Lean M.E. (2017). Weight losses with low-energy formula diets in obese patients with and without type 2 diabetes: Systematic review and meta-analysis. Int. J. Obes..

[36] Stefan N., Haring H.U., Schulze M.B. (2018). Metabolically healthy obesity: The low-hanging fruit in obesity treatment?. Lancet Diabetes Endocrinol..

[37] Huang L., Trieu K., Yoshimura S., Neal B., Woodward M., Campbell N.R.C., Li Q., Lackland D.T., Leung A.A., Anderson C.A.M. (2020). Effect of dose and duration of reduction in dietary sodium on blood pressure levels: Systematic review and meta-analysis of randomised trials. BMJ.

[38] Röhling M., Strom A., Bonhof G., Puttgen S., Bodis K., Mussig K., Szendrödi J., Markgraf D., Lehr S., Roden M. (2017). Differential Patterns of Impaired Cardiorespiratory Fitness and Cardiac Autonomic Dysfunction in Recently Diagnosed Type 1 and Type 2 Diabetes. Diabetes Care.

[39] Röhling M., Strom A., Bönhof G.J., Roden M., Ziegler D. (2017). Cardiorespiratory Fitness and Cardiac Autonomic Function in Diabetes. Curr. Diabetes Rep..

[40] Bhanpuri N.H., Hallberg S.J., Williams P.T., McKenzie A.L., Ballard K.D., Campbell W.W., McCarter J.P., Phinney S.D., Volek J.S. (2018). Cardiovascular disease risk factor responses to a type 2 diabetes care model including nutritional ketosis induced by sustained carbohydrate restriction at 1 year: An open label, non-randomized, controlled study. Cardiovasc. Diabetol..

