# Supplementary material for: A High-Protein and Low-Glycemic Formula Diet Improves Blood Pressure and Other Hemodynamic Parameters in High-Risk Individuals
Source: Nutrients. 2022 Mar 30;14(7):1443. doi: 10.3390/nu14071443 (PMC9003071; doi:10.3390/nu14071443)
Supplement: Supplementary file 1 [file nutrients-14-01443-s001.zip › nutrients-1599652-supplementary.pdf]

Table S1. Antihypertensive drugs of study completers at baseline compared between INT and CON

	INT-group (n=205)	CON-group (n=99)	P (INT vs. CON)
ACE inhibitors/ARBs (%)	38.9	32.7	0.285
Diuretics (%)	13.4	14.9	0.732
β blockers (%)	16.2	18.8	0.565
Calcium channel blockers (%)	9.3	11.9	0.470

Data are presented as percentages. ACE, angiotensin converting enzyme; ARB, angiotensin receptor blocker

Table S2. Correlation of changes in weight and blood pressure (SBP, DBP) stratified by the initial hypertensive, prehypertensive, or normotensive status

	Weight change [kg]	Change in SBP [mmHg]	Correlation / P value	Change in DBP [mmHg]	Correlation / P value
Follow-up after 6 months					
Hypertension<br>INT<br>CON	-8.7 [-11.1; -6.4]<br>-5.3 [-8.5; -2.1]	-16 [-23; -9]<br>-21 [-31; -12]	**0.542 / 0.003**<br>0.409 / 0.130	-7 [-9; -5]<br>-5 [-8; -2]	**0.365 / 0.050**<br>**0.595 / 0.019**
Prehypertension<br>INT<br>CON	-7.7 [-9.0; -6.4]<br>-4.4 [-6.5; -2.4]	-8 [-11; -5]<br>-5 [-10; -1]	**0.319 / 0.002**<br>0.134 / 0.417	-4 [-6; -1]<br>2 [-2; 5]	0.195 / 0.205<br>-0.021 / 0.900
Normotension<br>INT<br>CON	-5.9 [-7.2; -4.7]<br>-3.1 [-4.8; -1.4]	0 [-2; 2]<br>5 [2; 9]	0.005 / 0.294<br>0.108 / 0.480	-1 [-4; 2]<br>1 [-4; 5]	**0.287 / 0.006**<br>0.120 / 0.431
Follow-up after 12 months					
Hypertension<br>INT<br>CON	-6.1 [-8.4; -3.7]<br>-5.1 [-8.3; -1.8]	-17 [-26; -9]<br>-16 [-28; -5]	0.282 / 0.154<br>0.260 / 0.370	-5 [-7; -3]<br>4 [1; 7]	0.211 / 0.121<br>-0.119 / 0.686
Prehypertension<br>INT<br>CON	-5.3 [-6.7; -3.9]<br>-3.6 [-5.8; -1.4]	-6 [-9; -3]<br>0 [-5; 5]	**0.267 / 0.009**<br>0.072 / 0.657	-3 [-5; -1]<br>2 [-1; 6]	0.145 / 0.160<br>0.178 / 0.572
Normotension<br>INT<br>CON	-4.1 [-5.5; -2.8]<br>-2.9 [-4.8; -1.1]	3 [1; 6]<br>-2 [-6; 1]	0.101 / 0.343<br>0.128 / 0.406	2 [-1; 5]<br>3 [-2; 8]	0.101 / 0.257<br>0.131 / 0.398

Data are shown as mean [95% CI]. DBP, diastolic blood pressure; SBP, systolic blood pressure

Table S3. All contributors from the ACOORH Study Group

Name	Affiliation
Aloys Berg	Faculty of Medicine, University of Freiburg, Freiburg, Germany
Winfried Banzer	Department of Sports Medicine, Institute for Sports and Sport Science, University of Frankfurt, Frankfurt, Germany
Klaus Michael Braumann	Department of Sports and Movement Medicine, Faculty of Psychology and Human Movement Sciences, University of Hamburg, Hamburg, Germany
Martin Halle	Department of Prevention, Rehabilitation and Sports Medicine, Klinikum rechts der Isar, Technical University of Munich (TUM), Munich, Germany
Dagmar Führer-Sakel	Department of Endocrinology, Diabetes and Metabolism and Division of Laboratory Research, University Hospital Essen, University Duisburg-Essen, Essen, Germany
Kerstin Kempf	West-German Center of Diabetes and Health, Düsseldorf Catholic Hospital Group, Düsseldorf, Germany
Sadaf Koohkan	Faculty of Medicine, University of Freiburg, Freiburg, Germany
Stephan Martin	West-German Center of Diabetes and Health, Düsseldorf Catholic Hospital Group, Düsseldorf, Germany
David McCarthy	Public Health Nutrition Research Group, London Metropolitan University, London, UK
Michel Pinget	Department Endocrinologie, Diabete et Maladies Métaboliques, Faculte de Medicine de'l University de Strasbourg, Strasbourg, France
Hans Georg Predel	Institute of Cardiovascular Research and Sports Medicine, German Sport University Cologne, Cologne, Germany
Martin Röhling	West-German Center of Diabetes and Health, Düsseldorf Catholic Hospital Group, Düsseldorf, Germany
Nina Schaller	Department of Prevention, Rehabilitation and Sports Medicine, Klinikum rechts der Isar, Technical University of Munich (TUM), Munich, Germany
Isabelle Schenkenberger	KARDIOS, Cardiologists in Berlin, Berlin, Germany
Jürgen Scholze	KARDIOS, Cardiologists in Berlin, Berlin, Germany
Andrea Stensitzky	Faculty of Medicine, University of Freiburg, Freiburg, Germany
Susanne Tan	Department of Endocrinology, Diabetes and Metabolism and Division of Laboratory Research, University Hospital Essen, University Duisburg-Essen, Essen, Germany
Hermann Toplak	Department of Medicine, Division of Endocrinology and Diabetology, Medical University of Graz, Graz, Austria

Figure S1.

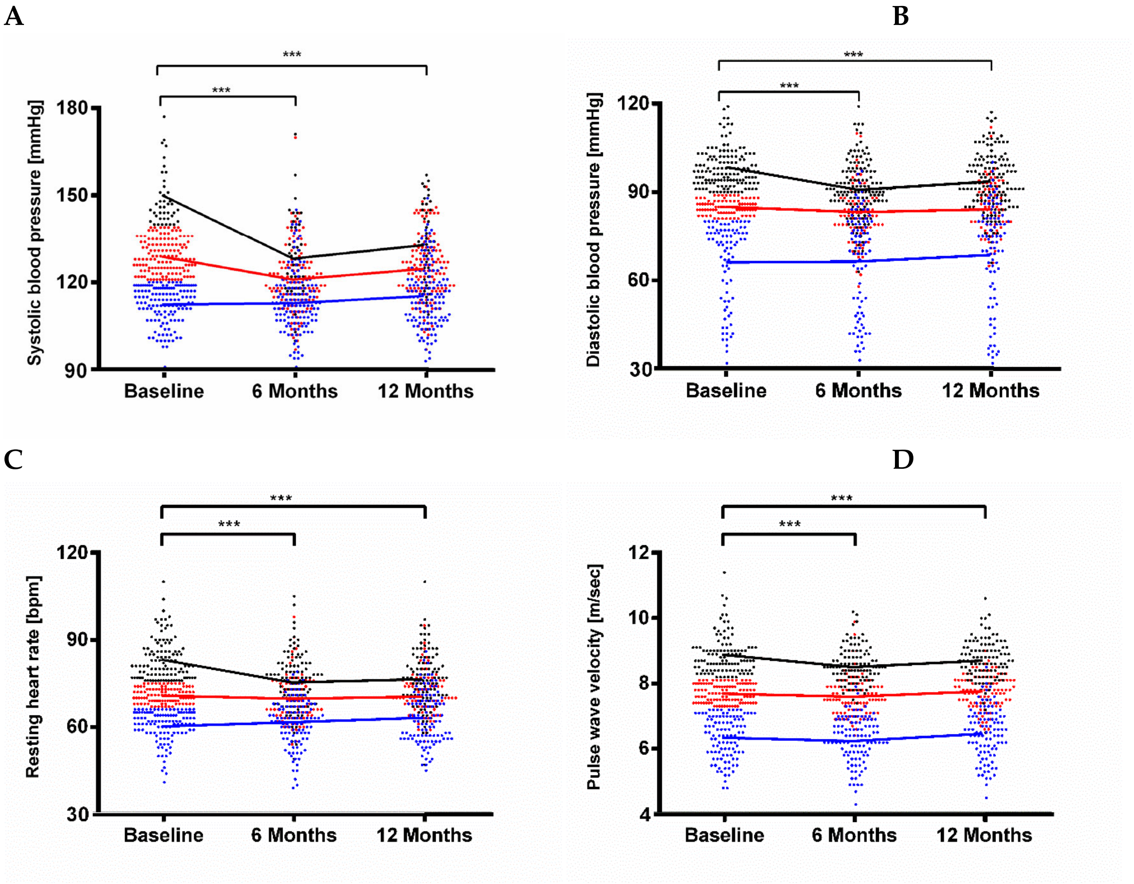

Figure S1. Changes of (A) SBP, (B) DBP, (C) HR, and (D) PWV after 6 and 12 months of all participants who finished the study (INT + CON). (A) Study cohort was partitioned in three groups according to the initial systolic blood pressure, namely hypertensive (black dots, ≥140 mmHg, n=43), prehypertensive (red dots, 121-139 mmHg, n=131), or normotensive (blue dots, ≤120 mmHg, n=130) state at baseline. Both, hypertension and prehypertension group reduced systolic blood pressure after 6 and 12 months; ***p<0.001 vs. baseline. (B) Study cohort was partitioned into three groups according to the initial diastolic blood pressure regarding a hypertensive (black dots, ≥90 mmHg, n=135), prehypertensive (red dots, 81-89 mmHg, n=84), or normotensive (blue dots, ≤80 mmHg, n=85) state at baseline. Both, hypertension and prehypertension group reduced diastolic blood pressure after 6 and 12 months; ***p<0.001 vs. baseline. (C) Study cohort was partitioned into three equally sized groups (tertiles) according to the initial resting heart rate (black dots – upper (3rd) tertile, n=103; red dots – middle (2nd) tertile, n=94; blue dots – lower (1st) tertile, n=107) at baseline. Both, upper and middle tertiles reduced resting heart rate after 6 and 12 months; ***p<0.001 vs. baseline. (D) Study cohort was partitioned into three equally sized groups (tertiles) according to the initial pulse wave velocity (black dots – upper (3rd) tertile, n=100; red dots – middle (2nd) tertile, n=102; blue dots – lower (1st) tertile, n=102) at baseline. Upper tertile reduced pulse wave velocity after 6 and 12 months; ***p<0.001 vs. baseline.

Figure S2

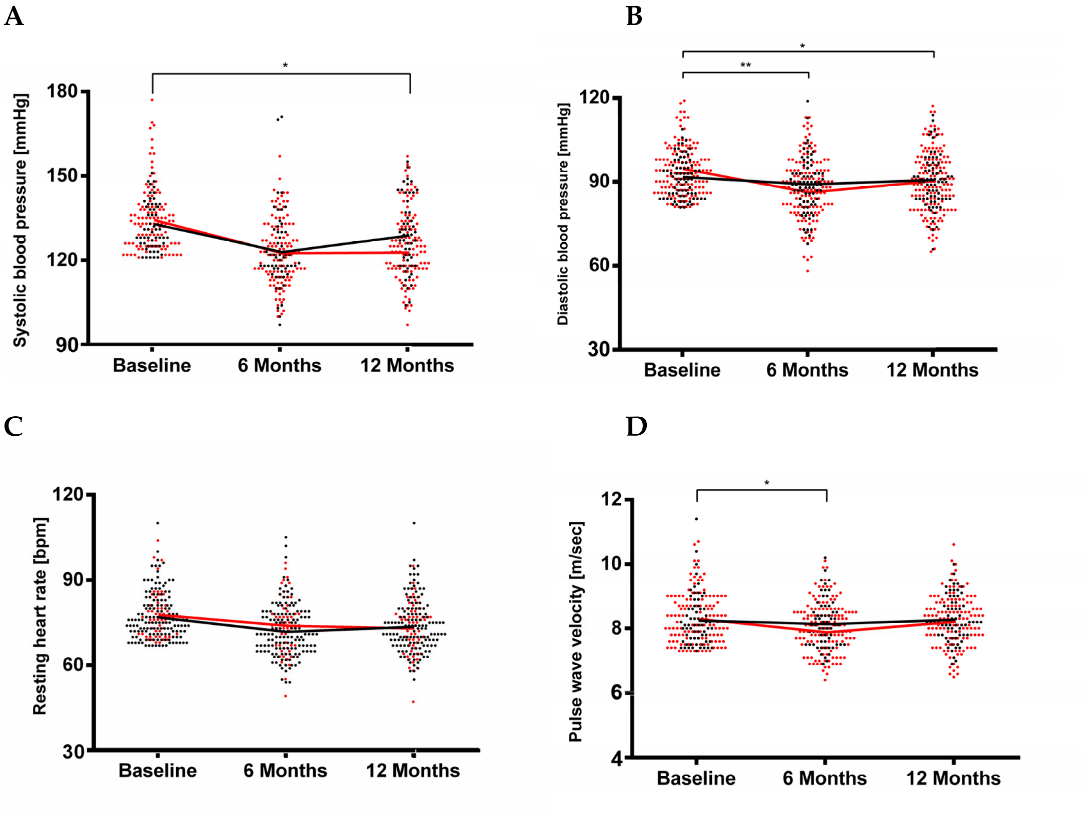

FiguresS2. Changes of (A) SBP, (B) DBP, (C) resting HR, and (D) PWV after 6 and 12 months compared between high-risk individuals of INT and CON. (A, B) Hypertensive and prehypertensive participants were examined for inter-group effects based on the group assignment to INT (red dots, formula diet + lifestyle intervention (SBP, n=119; DBP, n=146)) or CON (black dots, lifestyle intervention alone (SBP, n=55; DBP, n=73)). **p<0.01 INT vs CON; *p<0.05 INT vs CON;
(C, D) Upper and middle tertiles of resting HR and PWV were examined for inter-group effects based on the group assignment to INT (red dots, formula diet + lifestyle intervention (resting HR, n=137; PWV, n=147)) or CON (black dots, lifestyle intervention alone (resting HR, n=60; PWV, n=65)); *p<0.05
